# Acute Abdominal Pain Secondary to Omental Infarction: A Case Report

**DOI:** 10.7759/cureus.67694

**Published:** 2024-08-24

**Authors:** Sergio E Vázquez-Lara, Judith F Nava, Fausto G Nava-Garza, Miguel Jimenez-Yarza, Luis G Menchaca-Ramos

**Affiliations:** 1 General Surgery, Instituto de Seguridad y Servicios Sociales de los Trabajadores del Estado (ISSSTE) Regional Hospital, Monterrey, MEX; 2 Dermatology, Instituto Dermatologico de Jalisco, Zapopan, MEX

**Keywords:** emergency abdominal surgery, ct scan, omentum, acute abdominal pain, omental infarction

## Abstract

Omental infarction is a rare but threatening cause of acute abdomen. The preoperative diagnosis is challenging due to its infrequent nature. It poses nonspecific abdominal signs that can be easily mistaken for other more common intra-abdominal pathologies. Here, we report a case of a 31-year-old female who presented with acute abdominal pain. A simple CT scan of the abdomen showed signs suggestive of an omental infarction. An exploratory laparotomy was performed with resection of the mass, and histopathology reports confirmed the diagnosis. The diagnosis of omental infarction is complicated and rarely made prior to surgery. Surgical treatment provides better results and prevents complications.

## Introduction

Acute abdominal pain is a common complaint in patients presenting to the emergency department; approximately 4% to 6% of the patients refer to it as their main reason for consultation. Likewise, 10% of these patients are diagnosed with acute appendicitis (14%), intestinal obstruction (13%), urinary tract disorders (9%), acute diverticulitis (8%) and cholecystitis (5%) being the most frequent causes [1]. Abdominal pain can appear in different areas depending on the location of the omental infarction. Therefore, it is important to distinguish this condition from pathologies such as cholecystitis, appendicitis, diverticulitis, epiploic appendagitis and gynecological disorders [2]. Although about 400 cases of omental infarction have been reported so far, the exact incidence has not yet been determined. Approximately 85% of all cases have been observed in adults; it is more frequent in the age group of 40 to 50 years, with a male to female incidence of 2:1 [3]. Herein, we report a case of omental infarction with the aim of discussing its diagnosis and management and also adding information to the current literature.

## Case presentation

**Figure 1:**
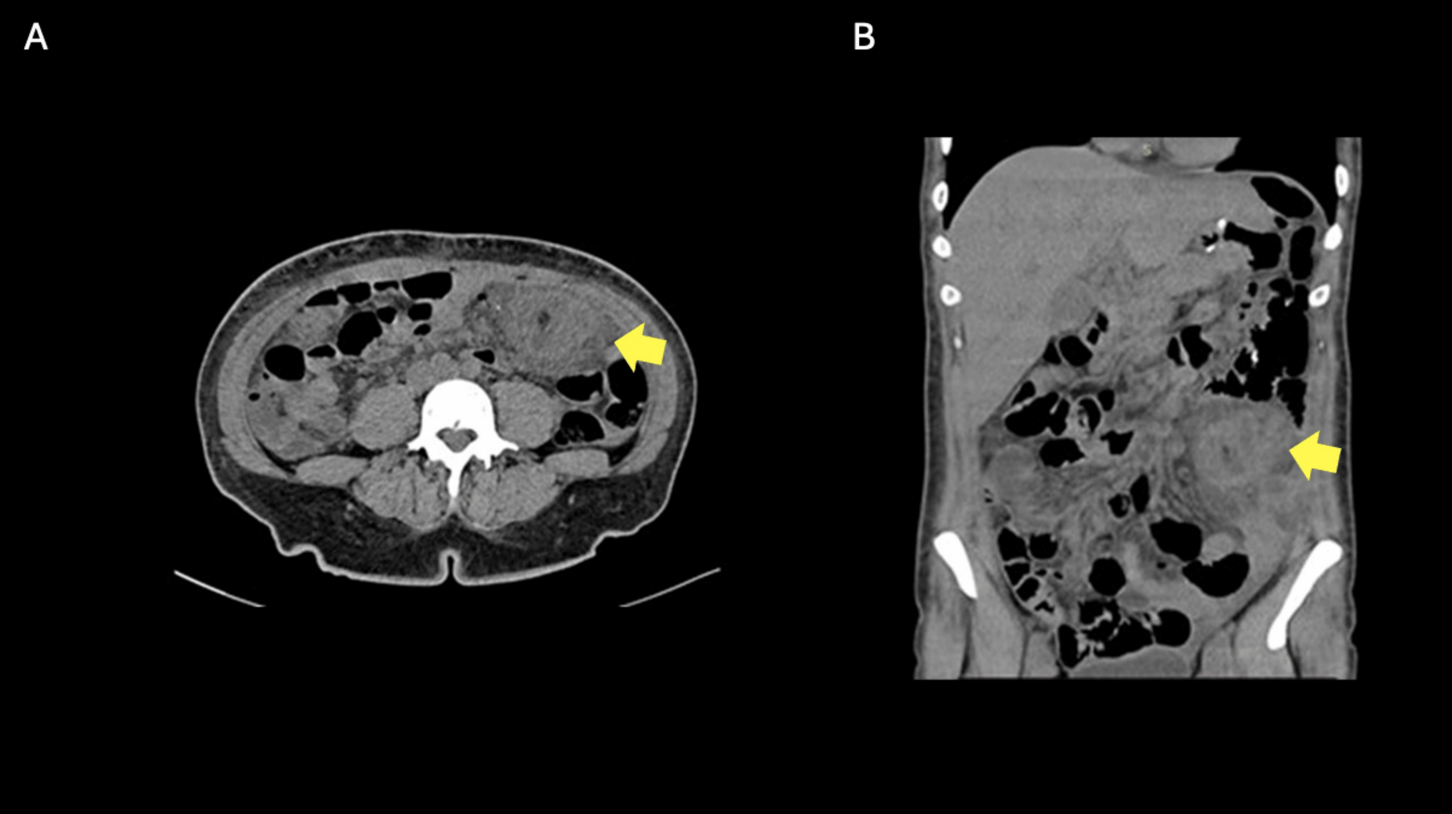
CT of the abdomen shows the circumscribed area with the inflammation centered around the omental fat at the time of diagnosis (A, axial; B, coronal view) The yellow arrow points to the area of omental infarction.

A 31-year-old female presented with colic abdominal pain in the epigastric region radiating to the left iliac fossa; the pain started three days prior to her admission to the emergency department. She had a history of a gastric bypass surgery done two years ago. A physical examination revealed decreased peristalsis with distended abdomen, depressible and painful to superficial palpation, presenting a mass in the left abdominal flank region. Laboratory studies showed leukocytes at 9.54 × 10^3^/μL and 77% neutrophils. A CT scan of the abdomen showed an acute inflammatory process at the level of the left omentum characterized by increased density and striation of the fat adjacent to the circumscribed lesion with a peripheral hyperdense border, all suggestive of an omental infarction (Figure 1).

**Figure 2:**
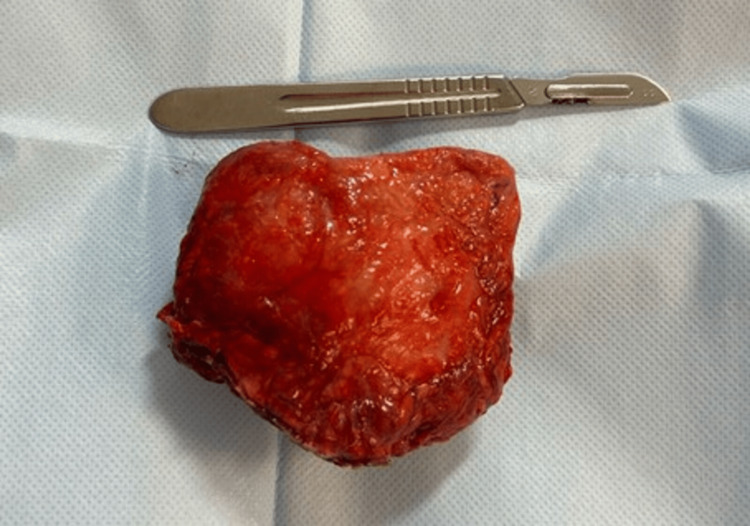
Resected specimen of the ischemic portion of the greater omentum

An exploratory laparotomy was performed; during the procedure, an omentum plastron was found in the left flank, showing early signs of ischemia. Subsequently, traction, exteriorization, resection, and hemostasis were effectuated with LigaSure (Figure 2).

**Figure 3:**
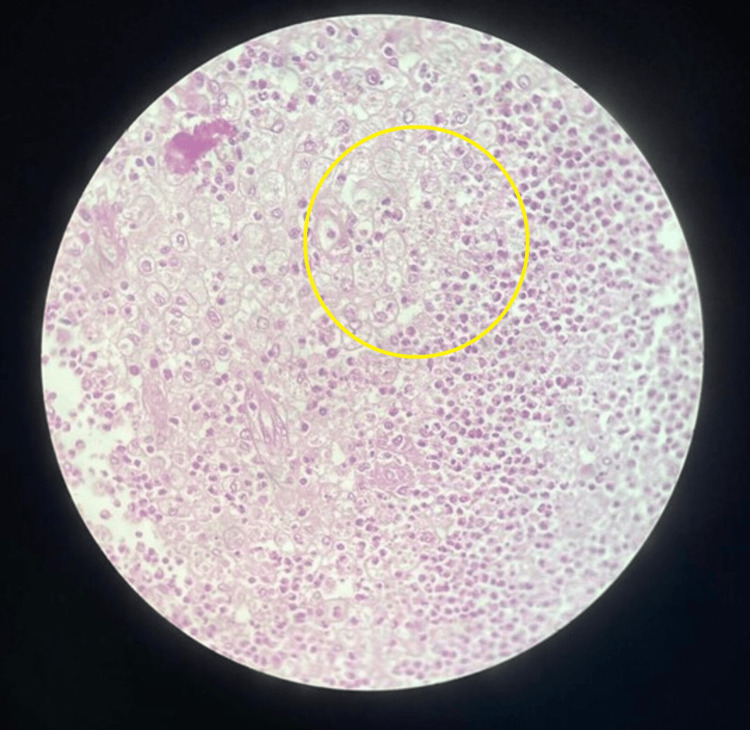
Fat necrosis with acute fibrinopurulent inflammation and abscess formation (circle)

Afterwards, we sent the specimen to pathology, which revealed fat necrosis, acute fibrinopurulent inflammation, and the formation of an abscess (Figure 3).

During the postoperative period, the patient had a satisfactory clinical outcome with no symptomatology. Control studies were requested showing leukocytes at 6.58 × 10^3^/μL and 42% neutrophils. She was discharged on her second day of hospital stay.

## Discussion

To our knowledge, about 400 cases of omental infarction have been reported so far, although the exact incidence has not yet been determined. This pathology is a rare cause of acute abdominal pain. Depending on the origin, this pathology can be classified as primary or secondary [4]. Primary or idiopathic omental infarction does not have a clearly established etiology. Some authors suggest the presence of anatomical variations as a possible cause [5]. On the other hand, secondary omental infarction can be caused by epiploic torsion, adhesions, inflammation, cysts, tumors, hernias, thrombosis, abdominal trauma, obesity, and right heart failure, among others [6]. Omental infarction induces venous stasis and thrombosis, which results in edema and congestion with hemorrhagic necrosis, and extravasation of serosanguineous peritoneal fluid. It has been described that omental infarction occurs more frequently on the right side. This may be because the omentum is longer and more mobile in that area compared to the left side [7]. Patients usually present with acute abdominal pain, nausea, vomiting, anorexia, fever and intestinal dysfunction. Laboratory studies show an increase in the leukocyte count and slightly elevated C-reactive protein levels [8]. Since these findings are unspecific, it is common to misdiagnosis it as appendicitis, cholecystitis or right diverticulitis. Therefore, computed tomography is a useful tool that helps us to distinguish this pathology among the main differential diagnoses. The findings are described as a well-circumscribed, oval peritoneal fat-attenuating mass and surrounding inflammatory changes with soft tissue strands that probably correspond to fibrous bands and/or dilated thrombosed veins. In addition, the mass is usually located deep in relation to the rectus abdominis muscle and anterior to the colon [9,10]. Standard treatment for omental infarction has not yet been established. Conservative management includes analgesics and anti-inflammatory drugs. This disease has a self-limited course; however, complications such as intestinal obstruction, abscess formation and adhesions may occur [6]. Nevertheless, surgical treatment not only accelerates recovery and provides better pain control, but also helps prevent complications [11]. Recently, this approach has been improved using the diagnostic laparoscopy, extending its application to therapeutic intervention.

## Conclusions

Due to its nonspecific presentation, the diagnosis of omental infarction is complicated and rarely made prior to surgery. Computed tomography provides a rapid and accurate diagnosis, making it possible to distinguish this condition from other causes of acute abdominal pain. Therefore, imaging diagnosis of omental infarction can help to avoid unnecessary surgical interventions. Although some patients recover spontaneously without surgery, segmental omentectomy is recommended to reduce the risk of subsequent complications. Histopathology results reporting a necrotic mass along with the presence of fat tissue, vascular congestion, thrombosis and nonspecific inflammatory infiltration confirm the diagnosis.
